# Anticancer effect of myristicin on hepatic carcinoma and related molecular mechanism

**DOI:** 10.1080/13880209.2021.1961825

**Published:** 2021-08-19

**Authors:** Hailan Bao, Qi Muge

**Affiliations:** a College of Traditional Mongolian Medicine, Inner Mongolia University for Nationalities, Tongliao, China; b Mengxi Integrative Medicine Division of Respiratory and Critical Care Medicine, Affiliated Hospital of Inner Mongolia University for Nationalities, Tongliao, China

**Keywords:** PI3K/Akt/mTOR signalling pathway, EMT, migration, invasion, proliferation, apoptosis

## Abstract

**Context:**

Myristicin is a natural active compound that has inflammatory, antimicrobial and anti-proliferative properties. Yet, its effect on hepatic carcinoma has not been investigated.

**Objective:**

To explore the role and related molecular mechanism of myristicin in hepatic carcinoma *in vitro*.

**Materials and methods:**

Human hepatic carcinoma cell lines (Huh-7 and HCCLM3 cells) were treated with different concentrations of myristicin (0.5, 1 and 5 mM) for 24, 48 and 72 h. Then, (3-(4,5-dimethylthiazol-2-yl)-2,5-diphenyltetrazolium bromide) tetrazolium assay (MTT), flow cytometer (FCM) analysis and transwell assay were performed to determine cell proliferation, apoptosis and migration/invasion, respectively. Protein levels of B-cell lymphoma-2 (Bcl-2), Bcl-2 associated X (Bax), E-cadherin, N-cadherin and phosphatidylinositol 3-kinase (PI3K)/protein kinase B (AKT)/mammalian target of rapamycin (mTOR) signalling pathway-related proteins were detected using Western blot assay. Gene expression was determined using quantitative real time-polymerase chain reaction (qRT-PCR).

**Results:**

Myristicin inhibited cell proliferation and induced apoptosis in Huh-7 and HCCLM3 cells; suppressed cell migration and invasion ability, and increased E-cadherin expression and decreased N-cadherin expression, thereby inhibiting epithelial–mesenchymal transition (EMT). Finally, the findings indicated that myristicin decreased phosphorylated (p)-mTOR and p-AKT expression at the protein level.

**Discussion and conclusions:**

Myristicin exerts an efficient therapeutic effect on hepatic carcinoma by suppressing PI3K/Akt/mTOR signalling pathway; thus, it may be used as a new potential drug for hepatic carcinoma treatment.

## Introduction

Hepatic carcinoma is one of the most common malignant tumours in China and the third most common cause of cancer death worldwide (Chedid et al. 2017; Hartke et al. 2017). It originates in liver cells or intrahepatic gallbladder epithelial cells, and there are obvious regional differences in its distribution. The main features of advanced hepatic carcinoma include liver pain, keratitis, jaundice, ascites and some other symptoms (El Jabbour et al. 2019). It is an aggressive type of tumour with a high rate of metastasis and recurrence that presents with no early symptoms; thus, its early diagnosis may be challenging, and its prognosis is poor (Yang et al. 2017). More than one million new cases are detected each year, among which 700,000 do not survive (Bray et al. 2018; Siegel et al. 2019). The increasing incidence has been found among 45–65 years old; yet, with the passing of time and the development of society, the incidence of hepatic carcinoma has been increasing among younger generations (FaRazi and Depinho 2006). The main treatments include tumour resection, chemotherapy and radiotherapy. However, the five-year survival rate of hepatic carcinoma patients remains low, especially due to drug resistance.

Myristicin (1-allyl-5-methoxy-3,4-methylenedioxybenzene) is a natural alkenylbenzene compound found in nutmeg (Lee et al. 2005; Martins et al. 2011), and in many favoured foods and dietary supplements, such as fennel, cinnamon, cloves, fennel, coriander, star anise and dried celery (Hallström and Thuvander 1997), and in some medicinal plants, such as *Todaroa aurea* (Sol.) Parl. (Apiaceae), *Daucus glochidiatus* (Labill.) Fisch. & C.A.Mey. (Apiaceae) and *Pseudorlaya pumila* (L.) Grande (Apiaceae). Previous research showed that myristicin possesses anti-inflammatory, antimicrobial and anti-proliferative properties (Lee and Park 2011; Stefano et al. 2011). In traditional medicine, myristicin has been used to treat cholera, stomach cramps, nausea, diarrhoea and anxiety (Martins et al. 2011). The inhibitory effects of myristicin on tumorigenesis have also been reported (Zheng et al. 1992; Lee et al. 2005). In this study, we explored the anticancer effect of myristicin on hepatic carcinoma and analysed the underlying regulatory mechanism.

## Materials and methods

### Cells

Human hepatic carcinoma cell (HCC) line Huh7 and HCCLM3 were obtained from American Tissue Culture Collection (ATCC). Cells were cultured in Dulbecco's modified Eagle medium (DMEM; Gibco, Grand Island, NY) medium supplemented with 10% foetal bovine serum (FBS; Gibco, Grand Island, NY) and 1% penicillin/streptomycin (Gibco, Grand Island, NY) in a humidified atmosphere containing 5% CO_2_/95% air at 37 °C.

### MTT assay

Cells were cultured in a 96-well plate at a density of 1.5 × 10^4^ cells per well and treated with different concentrations of myristicin (0.5, 1 and 5 mM) (Lee et al. 2005) for 24, 48 and 72 h. Cells without myristicin treatment were considered as the control. At each time point, 20 μL of sterile MTT dye (5 mg/mL, Sigma, St. Louis, MO) was added to each well and incubated for another 4 h at 37 °C. The absorbance was measured at 570 nm by a microplate reader (Bio-Rad, Hercules, CA). The data were analysed as the means ± standard deviation (S.D.) of three separate experiments.

### FCM assay

Cell apoptosis was performed by using the annexin-V/propidium iodide (PI) Apoptosis Detection Kit (BD Biosciences, Franklin Lakes, NJ). Briefly, after cells were treated with different concentrations of myristicin (0.5, 1 and 5 mM) for 48 h. Cells without myristicin treatment were considered as the control. Cells were collected, centrifuged at low temperature at high speed, and resuspended in 100 μL of FITC-binding buffer. Subsequently, samples were mixed with 5 μL ready-to-use annexin V-FITC and 5 μL PI into the buffer for 30 min at room temperature in dark. Annexin V-FITC and PI fluorescence were assessed by BD FACSCalibur flow cytometer (BD Technologies, Franklin Lakes, NJ).

### Transwell assay

We used a transwell experiment to detect the cell migration and invasion ability. The difference between migration and invasion experiments was whether Matrigel (BD Biosciences, Franklin Lakes, NJ) was presented. Huh-7 or HCCLM3 cells were plated in a 24-wells plate and treated with myristicin for 48 h. Cells without myristicin treatment were considered as the control. Cells were then digested and resuspended in a serum-free DMEM medium. Next, they were placed in an upper chamber, while a DMEM medium with 20% FBS was placed in a lower chamber. After 24 h, the cells in the lower chamber were washed, fixed and then stained with 0.1% crystal violet. Finally, the invasive and migratory cells were observed with a microscope.

### Caspase-3 activity detection

We detect caspase-3 activity by using a caspase-3 activity detection kit (Beyotime Biotechnology, Shanghai, China). Briefly, cells were collected into an EP tube, centrifuged at 600×*g* for 5 min at 4 °C, and lysed for 15 min in an ice bath. After centrifugation for 10–15 min, the supernatant was collected and placed in an ice-bath pre-cooled centrifuge tube, after which the caspase-3 enzyme activity was measured at 405 nm.

### qRT-PCR assay

Total RNA was acquired using TRIzol (TaKara, Kusatsu, Japan) following the manufacturer’s instructions. The RNA was transformed into cDNA by reverse transcription using cDNA (Vazyme, Nanjing, China). Subsequently, cDNA was used for amplification. We performed qPCR with SYBR Green PCR kit (Vazyme, Nanjing, China) followed by reference manual. GAPDH was used as endogenous control. Primer sequences for PCR were listed as following:

GAPDH, forward: 5′-CTTTGGTATCGTGGAAGGACTC-3′;
reverse: 5′-GTAGAGGCAGGGATGATGTTCT-3′;
Bcl-2, forward: 5′-GATCCTCGAGATGGCGCACGCTGGGAGAAC-3′;
reverse: 5′-GATCGGATCCTCATGGCTGAGCGCAG-3′;
Bax, forward: 5′-GGACGAACTGGACAGTAACATGG-3′;
reverse: 5′-GCAAAGTAGAA-AAGGGCGACAAC-3′;
E-cadherin, forward, 5′-CGAGAGCTACACGTTCACGG-3′;
reverse: 5′-GGGTGTCGAGGGAAAAATAGG-3′;
N-cadherin, forward, 5′-TTTGATGGAGGTCTCCTAACACC-3′;
reverse: 5′-ACGTTTAACACGTTGGAAATGTG-3′.
  The $2^{-\Delta \Delta C_{T}}$ method (Livak and Schmittgen 2001) was used to quantify relative gene expression.

### Western blot assay

The cells were lysed, and total protein was obtained by using RIPA buffer (Solarbio, Beijing, China). A bicinchoninic acid assay kit (Pierce, Appleton, WI) was used to quantify the total protein. An equal number of proteins were separated by 12% sodium dodecyl sulphate polyacrylamide gel electrophoresis (SDS-PAGE) for 40 min and then transferred to polyvinylidene fluoride (PVDF) membranes. The membranes were blocked for 1.5 h with 5% non-fat milk to prevent non-specific binding and then incubated with primary antibodies including anti-B-cell lymphoma-2 (Bcl-2) (cat. no. ab32124; dilution: 1:1000; Abcam, Cambridge, UK), anti-E-cadherin (cat. no. ab227639; dilution: 1:1000; Abcam, Cambridge, UK), anti-N-cadherin (cat. no. ab76011; dilution: 1:1000; Abcam, Cambridge, UK), anti-mTOR (cat. no. ab134903; dilution: 1:1000; Abcam, Cambridge, UK), anti-p-mTOR (cat. no. ab137133; dilution: 1:1000; Abcam, Cambridge, UK), anti-AKT (cat. no. 9272; dilution: 1:1000; Cell Signaling Technology, Inc., Boston, MA), anti-p-AKT (cat. no. 4060; dilution: 1:1000; Cell Signaling Technology, Inc., Boston, MA) and anti-Bcl-2 associated X (Bax) (cat. no. ab32503; 1:1000, Abcam, Cambridge, UK) at 4 °C overnight, and then with horseradish peroxidase-conjugated anti-rabbit immunoglobulin G secondary antibody (cat no. 7074; 1:2,000; Cell Signaling Technology, Inc., Boston, MA) at room temperature for 2 h. The protein bands were visualized by the enhanced chemiluminescence method (GE Healthcare Life Sciences, Piscataway, NJ). GAPDH (1:1000, Abcam, Cambridge, UK) served as a loading control for normalization. Protein expression was quantified using ImageJ software (version 1.46; National Institutes of Health, Bethesda, MD).

### Statistical analysis

Results were analysed by GraphPad Prism 6.0 software (La Jolla, CA). Statistical significance between groups was determined by Student’s *t*-test or one-way analysis of variance (ANOVA) followed by Tukey’s *post hoc* tests. Data were from three independent experiments. A *p* < 0.05 was considered to be statistically significant.

## Results

### The effect of myristicin on HCC proliferation

**Figure 1.**
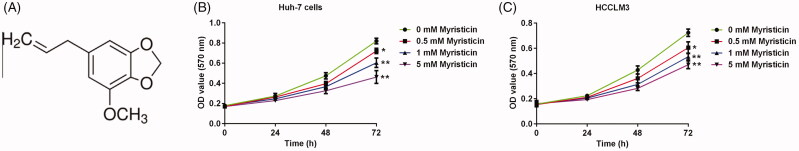
Myristicin suppresses HCC proliferation in a dose-dependent manner. (A) The chemical formula of myristicin. (B, C) MTT analysis revealing cell viability when Huh-7 cells and HCCLM3 cells were treated with 0.5, 1 and 5 mM myristicin for 24 h, 48 h and 72 h. *^,^**p < 0.05, 0.01 vs. 0 mM myristicin treatment group.

Myristicin is a natural alkenylbenzene compound. Its chemical formula is shown in Figure 1(A). MTT assay was used to explore the effect of myristicin on HCC viability. Our results showed that compared to the control group, myristicin significantly suppresses Huh-7 (Figure 1(B)) and HCCLM3 (Figure 1(C)) cell proliferation.

### The effect of myristicin on HCC apoptosis

**Figure 2.**
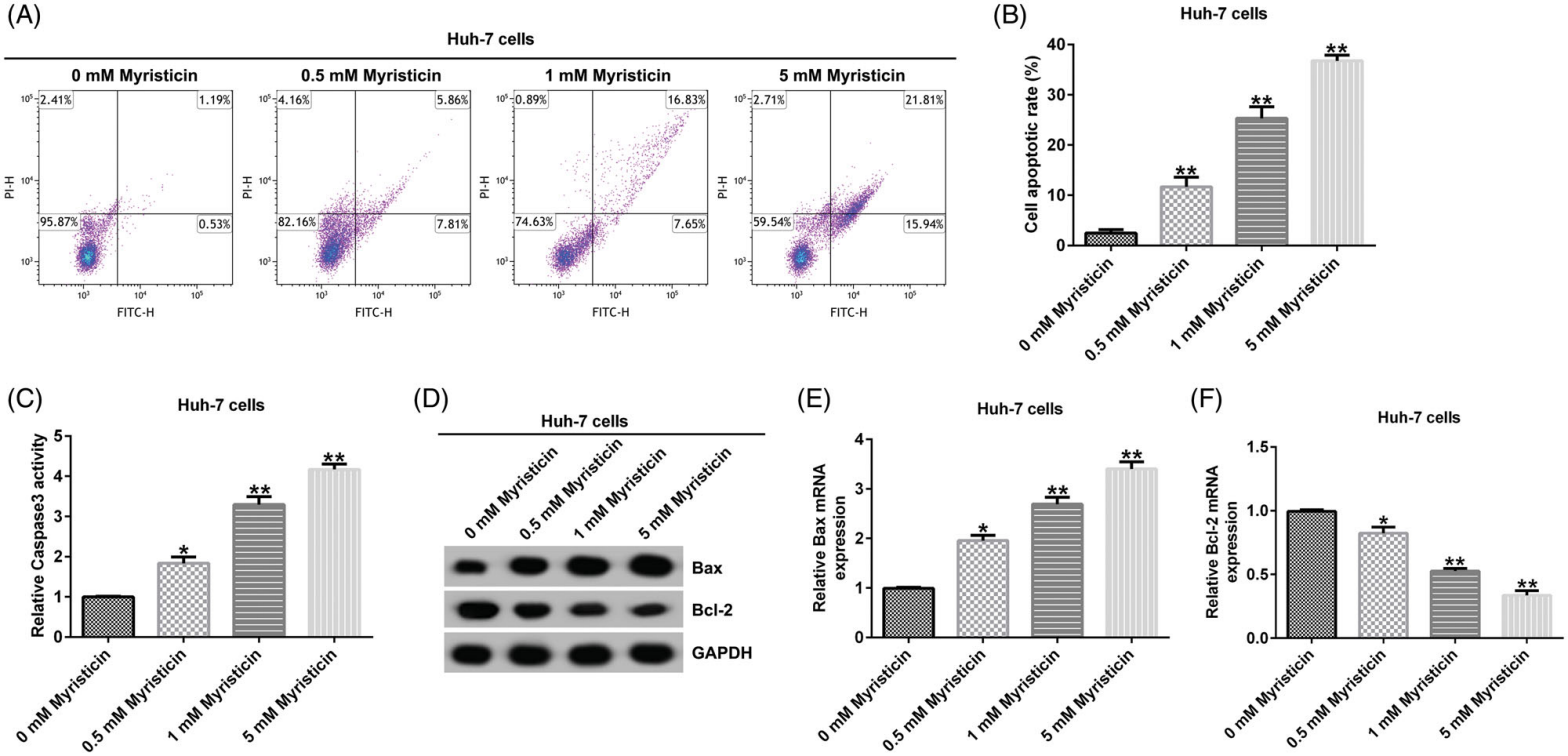
Myristicin induces cell apoptosis in Huh-7 cells in a dose-dependent manner. (A) FCM assay. (B) Cell apoptosis ratio analysed by a GraphPad Prism 6.0 software (La Jolla, CA). (C) Caspase3 activity was measured by the caspase3 detection kit. (D) Bcl-2 and Bax protein expression analysis by Western blot. (E, F) Bcl-2 and Bax mRNA expression analysis by qRT-PCR. *^,^**p < 0.05, 0.01 vs. 0 mM myristicin treatment group.

**Figure 3.**
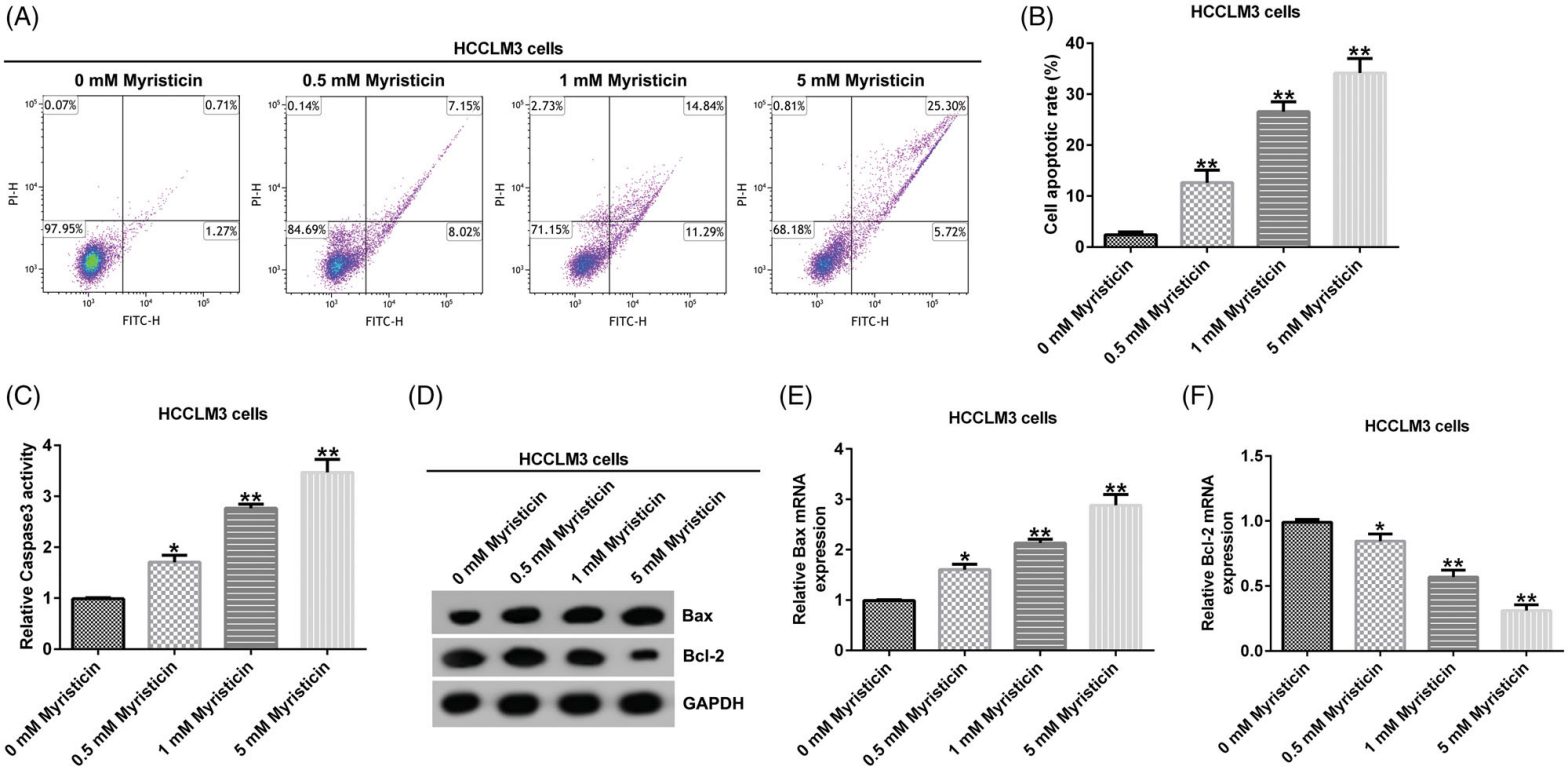
Myristicin induces cell apoptosis in HCCLM3 cells. (A) FCM assay. (B) Cell apoptosis ratio analysed by a GraphPad Prism 6.0 software (La Jolla, CA). (C) Caspase-3 activity was measured by the caspase-3 detection kit. (D) Bcl-2 and Bax protein expression analysis by Western blot. (E, F) Bcl-2 and Bax mRNA expression analysis by qRT-PCR. *^,^**p < 0.05, 0.01 vs. 0 mM myristicin treatment group.

The effect of myristicin on early and late apoptosis was examined using a flow cytometer (FCM) assay to detect cell apoptosis. FCM assay showed that compared to the control group, myristicin significantly induced apoptosis and improved caspase-3 activity in Huh-7 cells (Figure 2(A–C)) and HCCLM3 cells (Figure 3(A–C)). Meanwhile, myristicin decreased Bcl-2 expression and increased Bax expression at protein and mRNA levels in Huh-7 cells (Figure 2(D–F)) and HCCLM3 cells (Figure 3(D–F)). Taken together, myristicin promoted cell apoptosis.

### The effect of myristicin on the migration and invasion of HCC

**Figure 4.**
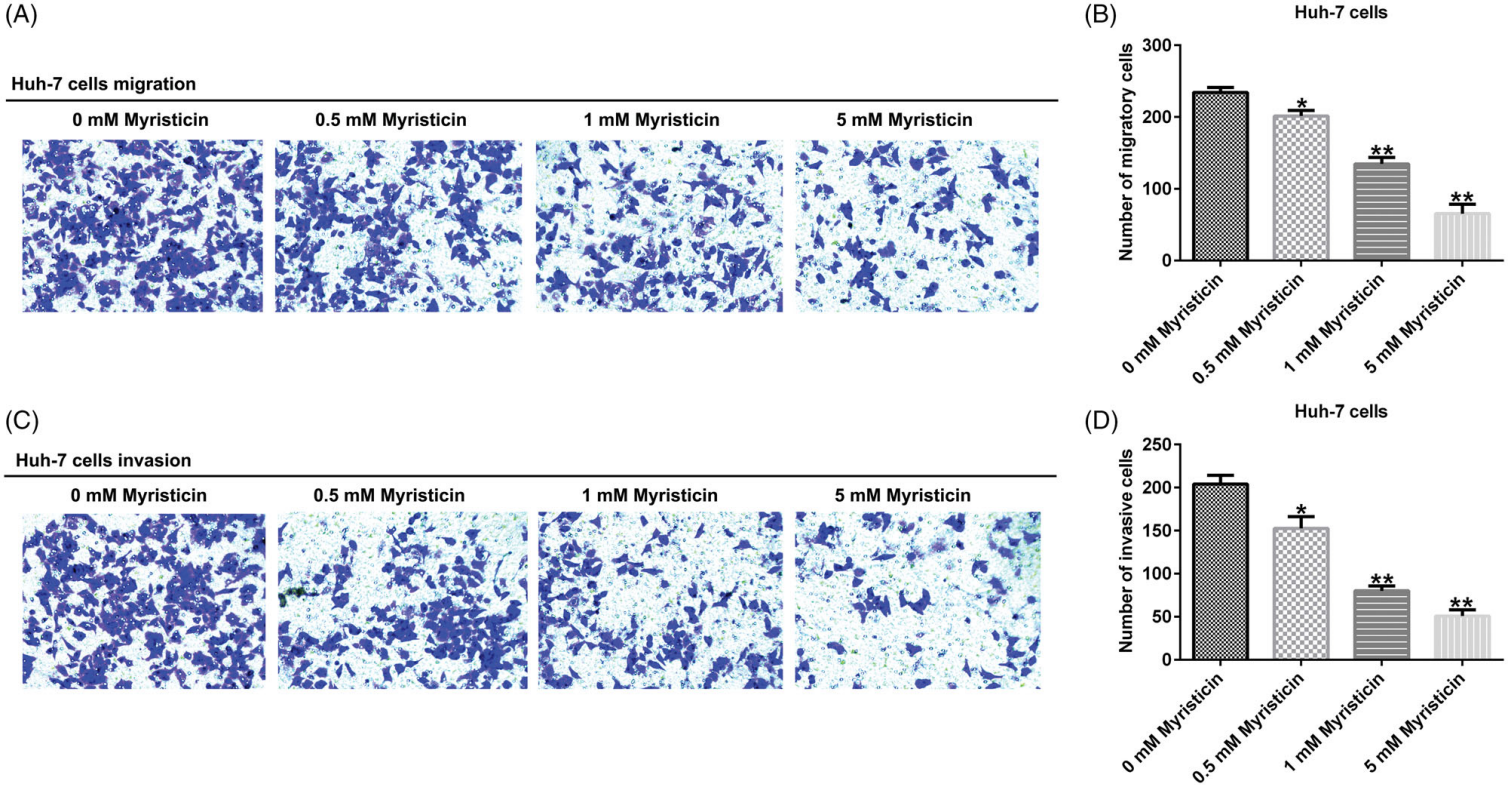
Myristicin suppressed migration and invasion of Huh-7 cells. (A) Transwell analysis of cell migration without matrigel in the lower chamber. (B) The number of migratory cells expressed as mean ± S.D. (C) Transwell analysis of cell invasion with matrigel in the lower chamber. (D) The number of invasive cells expressed as mean ± S.D. *^,^**p < 0.05, 0.01 vs. 0 mM myristicin treatment group.

**Figure 5.**
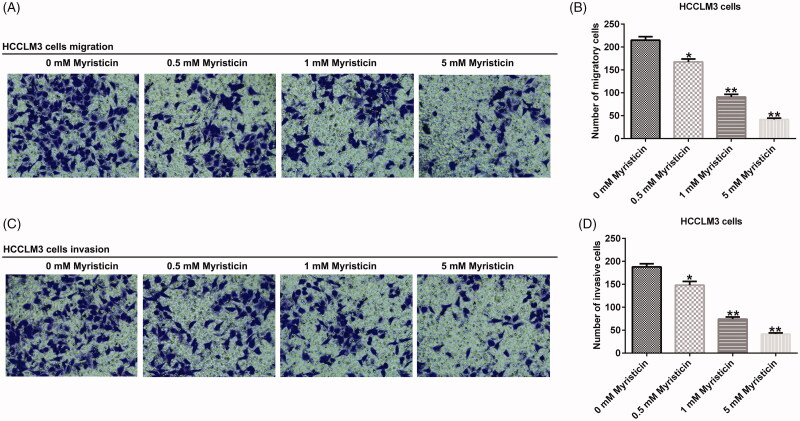
Myristicin suppressed migration and invasion of HCCLM3 cells. (A) Transwell analysis of HCCLM3 cell migration. (B) The number of migratory cells. (C) Transwell analysis of HCCLM3 cell invasion. (D) The number of invasive cells. *^,^**p < 0.05, 0.01 vs. 0 mM myristicin treatment group.

Our results demonstrated that with the increase of myristicin concentration, migration (Figure 4(A,B)) and invasion (Figure 4(C,D)) ability of Huh-7 cells was significantly inhibited compared with the control group. The same results were observed in HCCLM3 cells (Figure 5(A–D)).

### The effect of myristicin on EMT of HCC

**Figure 6.**
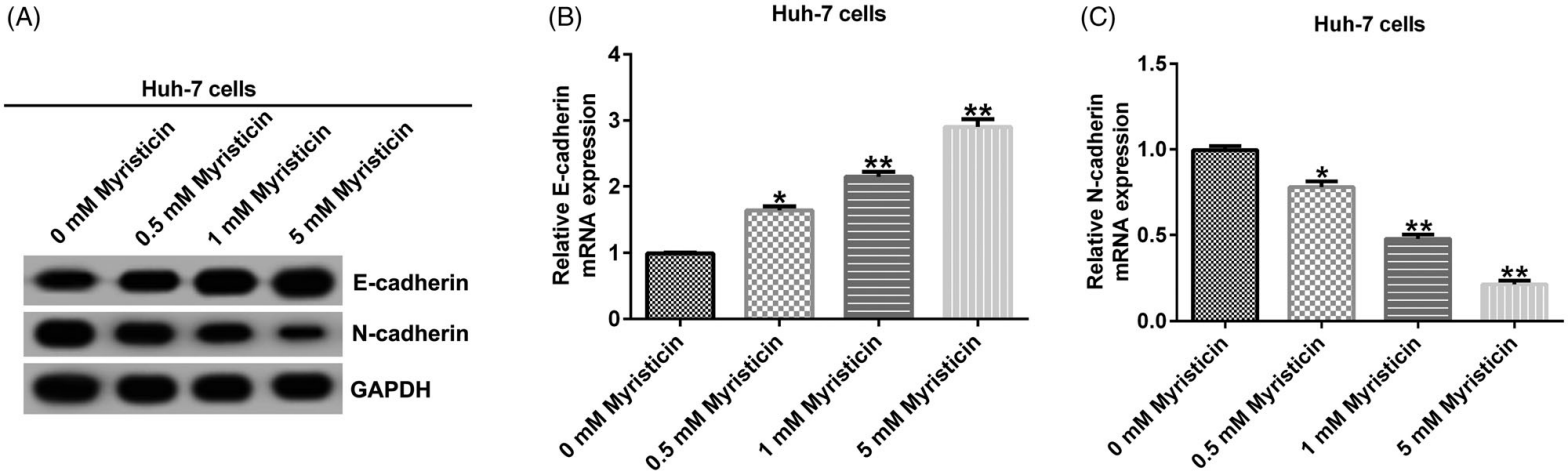
Myristicin inhibits EMT in Huh-7 cells. Huh-7 cells were treated with 0.5, 1 and 5 mM myristicin for 48 h. (A) Western blot analysis of E-cadherin and N-cadherin expression. (B) qRT-PCR analysis of E-cadherin expression. (C) qRT-PCR analysis of N-cadherin expression. *^,^**p < 0.05, 0.01 vs. 0 mM myristicin treatment group.

**Figure 7.**
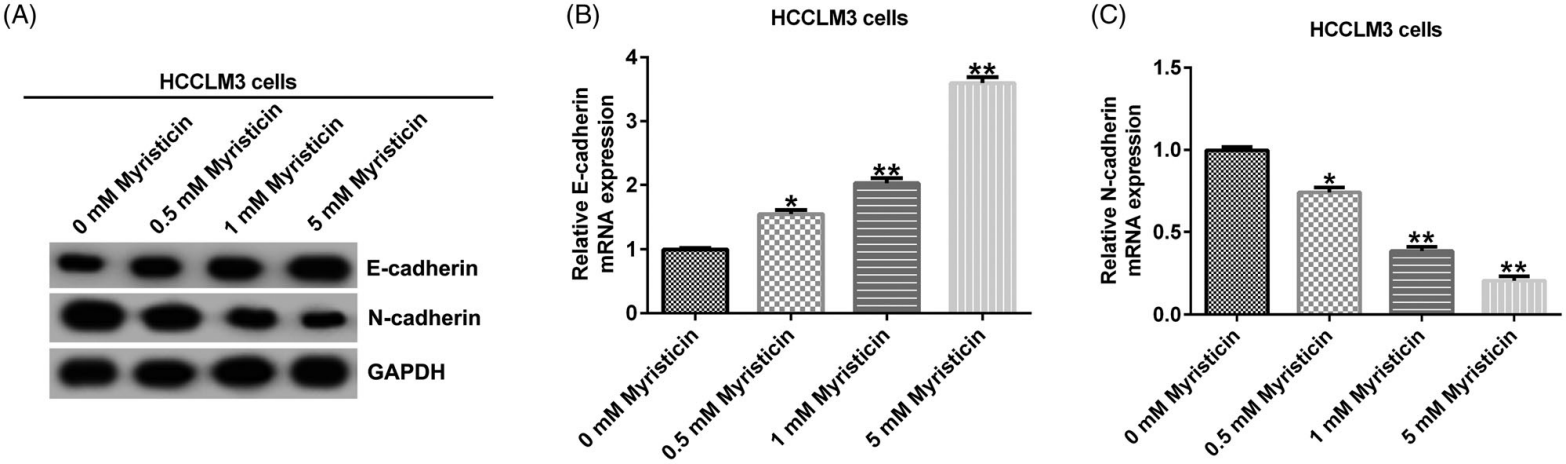
Myristicin inhibits EMT in HCCLM3 cells. (A) Western blot analysis of E-cadherin and N-cadherin expression. (B) qRT-PCR analysis of E-cadherin expression. (C) qRT-PCR analysis of N-cadherin expression. *^,^**p < 0.05, 0.01 vs. 0 mM myristicin treatment group.

Epithelial–mesenchymal transition (EMT) is an important sign of cancer metastasis. We detected EMT-related protein expression. Western blot assay indicated that compared to the control group, myristicin increased E-cadherin expression and decreased N-cadherin expression in HCC cells (Figures 6(A) and 7(A)). In addition, quantitative real time-polymerase chain reaction (qRT-PCR) assay showed that compared to the control group, E-cadherin was significantly up-regulated (Figures 6(B) and 7(B)) and N-cadherin was down-regulated (Figures 6(C) and 7(C)) in HCC cells.

### Myristicin suppressed PI3K/Akt/mTOR signalling pathway in HCC

**Figure 8.**
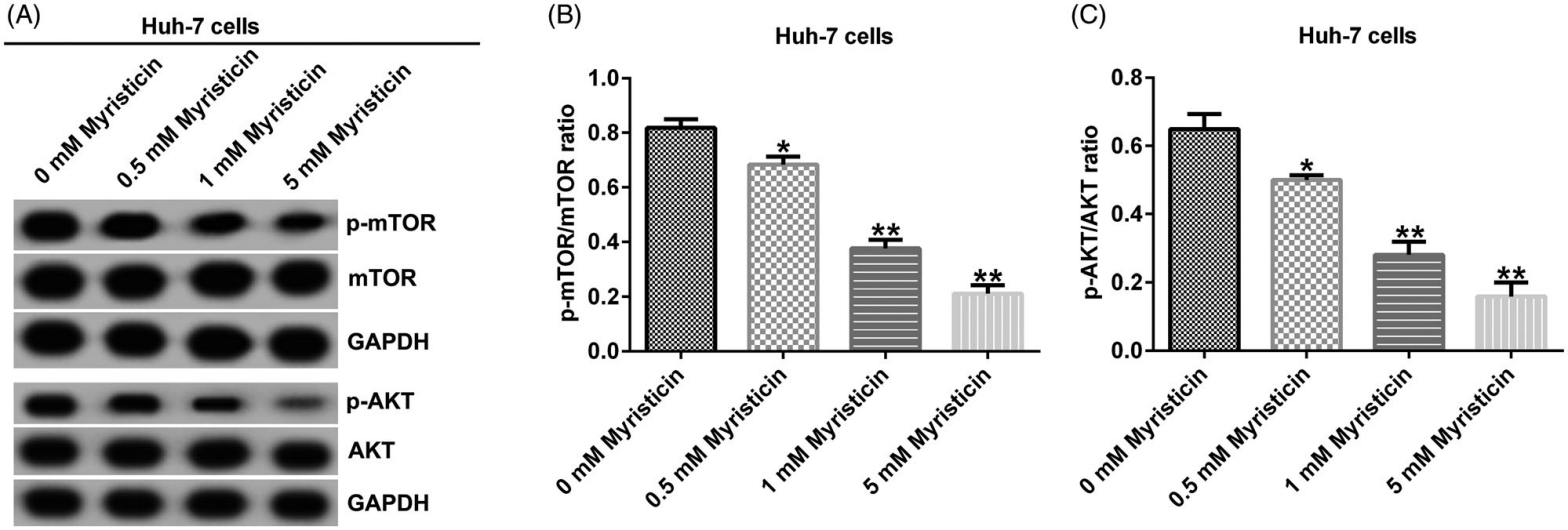
Myristicin suppresses the PI3K/Akt/mTOR signalling pathway in Huh-7 cells. (A) Western blot analysis of p-mTOR and p-AKT expression in Huh-7 cells. (B) The ratio of p-mTOR/mTOR in Huh-7 cells. (C) The ratio of p-AKT/AKT in Huh-7 cells. *^,^**p < 0.05, 0.01 vs. 0 mM myristicin treatment group.

**Figure 9.**
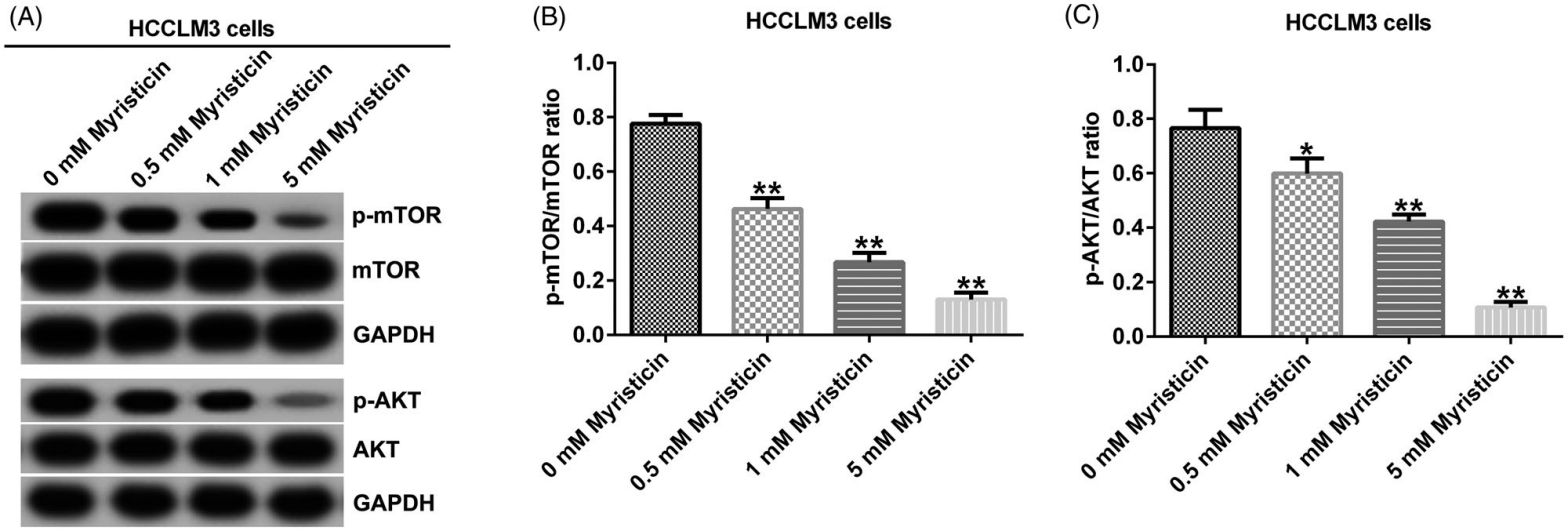
Myristicin suppresses the PI3K/Akt/mTOR signalling pathway in HCCLM3 cells. (A) Western blot analysis of p-mTOR and p-AKT expression in HCCLM3 cells. (B) The ratio of p-mTOR/mTOR in HCCLM3 cells. (C) The ratio of p-AKT/AKT in HCCLM3 cells. *^,^**p < 0.05, 0.01 vs. 0 mM myristicin treatment group.

To explore the mechanism of myristicin in HCC, we detected some protein expression in phosphatidylinositol 3-kinase (PI3K)/Akt/mTOR signalling pathway. Western blot assay showed that in Huh-7 and HCCLM3 cells, myristicin decreased p-mTOR and p-AKT expression (Figures 8(A) and 9(A)) and significantly decreased the ratio of p-mTOR/mTOR (Figures 8(B) and 9(B)) and p-AKT/AKT (Figures 8(C) and 9(C)) in HCC cells.

## Discussion

The development of hepatic carcinoma has seriously affected people’s living standards. In recent years, although the medical level has been continuously improved, hepatic carcinoma has a high mortality rate due to the difficulty of early diagnosis of hepatic carcinoma patients, fewer therapeutic targets and poor postoperative recovery. After surgical resection of hepatic carcinoma patients, the number of patients who survived one year after surgery was more than 80%, and the number of patients who survived 5 years was more than 50% (Margarit et al. 2005). Therefore, it is very important to continuously explore new treatment strategies to treat hepatic carcinoma patients. In the present study, we provided some evidence that myristicin is a natural compound that can be used for HCC treatment to a certain extent.

Myristicin is the main active ingredient of nutmeg (Jana and Shekhawat 2010; Muchtaridi et al. 2010) and coriander leaf oil (Zheng et al. 1992; Wei and Shibamoto 2007), which is mainly used as a flavouring agent, but also as a spice in the cosmetics industry. Previous research showed that myristicin could produce excitement and hallucinogenic effects. It may also be potentially toxic, as overdose can cause hallucinations and, in some cases, even coma (Stein et al. 2001; Sivathanu et al. 2014). Lee and Park (2011) demonstrated that myristicin has anti-inflammatory effects by inhibiting inflammatory factor expression. Lee et al. (2005) further found that myristicin induces early apoptotic events in human neuroblastoma, including cytochrome c release, caspase-3 activation and PARP lysis, leading to the death of SK-N-SH cells. In our study, we investigated the effect of myristicin on hepatic carcinoma. Our results showed that myristicin suppressed cell proliferation and induced apoptosis in Huh-7 and HCCLM3 cells.

Hepatic carcinoma is an aggressive type of tumour with a high rate of metastasis. As the continuous metastasis of tumours leads to high incidence and mortality, it is necessary to effectively treat hepatic carcinoma from the perspective of inhibiting cancer metastasis (Wu et al. 2015). Tumour metastasis depends on the migration and invasion capabilities of cancer cells (Hua et al. 2018). In this study, myristicin inhibited cell migration and invasion.

EMT is a process that refers to the transformation of epithelium to mesenchymal cells, which gives cells the ability to transfer and invade (Lamouille et al. 2014). EMT is currently considered an important reminder of cancer metastasis (Ye et al. 2017). In addition, during the early stage of cancer metastasis, the adhesion of cancer cells can be separated by reducing EMT. The main markers of EMT include epithelial cell marker (E-cadherin) and mesenchymal cell markers (N-cadherin). In this study, we found that myristicin suppressed EMT by increasing E-cadherin expression and decreasing N-cadherin expression.

PI3K/Akt/mTOR signalling pathway is involved in development of many cancers (Costa et al. 2018; Alzahrani 2019; Ediriweera et al. 2019). Activation of this signalling pathway can promote cell growth, proliferation and participate in angiogenesis (Alzahrani 2019). It can also exert a crucial role in mediating cell movement, invasion and metastasis (Jeong et al. 2014; Ramadan et al. 2020). PI3K/Akt/mTOR signalling pathway inhibition has been shown to be used to inhibit HCC (Kudo 2011; Ye et al. 2019). Hua et al. (2018) indicated that ruscogenin inhibits HCC migration and invasion by regulating PI3K/Akt/mTOR signalling pathway. Furthermore, Song et al. (2020) indicated that miR-29-3p overexpression suppresses HCC growth by inhibition of Robo1 and inactivation of PI3K/Akt/mTOR signalling pathway. Also, Sun et al. (2019) showed that miR-1914 exerted the inhibiting effect through the PI3K/Akt/mTOR signalling pathway in HCC. Consistent with the previous research results, our data suggested that myristicin suppresses the activation of the PI3K/Akt/mTOR signalling pathway in hepatic carcinoma.

However, this study is a preliminary *in vitro* study of the effect of myristicin on hepatocellular carcinoma. In order to make myristicin's effect on hepatocellular carcinoma more convincing, more in-depth research is needed. For example, the role of myristicin in other types of hepatic carcinoma cell lines should be studied. Besides, the effect of more doses of myristicin (a minimum of five doses is required) on HCC cells should be studied. Moreover, additional *in vivo* studies are required to confirm these findings. Therefore, in our next study, we plan to examine the effect of more dose of myristicin in other types of hepatic carcinoma cell lines and in a mouse model bearing an HCC tumour.

## Conclusions

Myristicin prevents the malignant biological behaviour of hepatic carcinoma cells by inhibiting the PI3K/Akt/mTOR signalling pathway. Thus, myristicin is a potential therapeutic agent for hepatic carcinoma.
